# Awe in Childhood: Conjectures About a Still Unexplored Research Area

**DOI:** 10.3389/fpsyg.2022.791534

**Published:** 2022-01-28

**Authors:** Claire Prade

**Affiliations:** Department of Developmental and Differential Psychology, Research Center for the Psychology of Knowledge, Language, and Emotion, Aix-Marseille University, Aix-en-Provence, France

**Keywords:** self-development, emotions in children, awe experiences, cognitive accommodation, self-awareness, self-transcendent emotions

Throughout his life, Albert Einstein would retain the intuition and the awe of a child. He never lost his sense of wonder at the magic of nature's phenomena-magnetic fields, gravity, inertia, acceleration, light beams-which grown-ups find so commonplace. He retained the ability to hold two thoughts in his mind simultaneously, to be puzzled when they conflicted, and to marvel when he could smell an underlying unity. “People like you and me never grow old,” he wrote a friend later in life. “We never cease to stand like curious children before the great mystery into which we were born.”

—Walter Isacsoon, Einstein: His Life and Universe

In his famous biography of Albert Einstein, Walter Isaacson shared the idea that awe might be a particularly important emotion during childhood, notably in the process of knowledge acquisition and learning. In the conclusion of a recent scientific publication, Valdesolo et al. (2017) made similar assumptions. Indeed, given that awe is triggered by a need for cognitive accommodation (Keltner and Haidt, 2003; Campos et al., 2013) and that cognitive accommodation underlies learning from an early age Piaget and Inhelder (1969/1969), Valdesolo et al. (2017) claimed that awe may be deeply involved in the process of early learning. The present article aims to specify this assumption. In a first part, features of awe and its differences with other epistemic emotions will be examined. Afterwards, conjectures about the emergence and development of awe in childhood will be made in order to understand the involvement of awe in early learning.

## Features of Awe

Awe has been theorized as the emotional response to strikingly vast or powerful stimuli that challenge the individuals' current frames of reference (Keltner and Haidt, 2003) and therefore cause them to rethink their understanding of the world (e.g., Saroglou et al., 2008; Büssing et al., 2021; Stancato and Keltner, 2021). In other words, two appraisals are needed to be met to experience awe: perception of vastness and a need for knowledge restructuring (Keltner and Haidt, 2003; Campos et al., 2013; Shiota et al., 2014).

Knowledge restructuring, also labeled cognitive accommodation, refers to the process of adjusting mental structures that cannot assimilate a new experience into preexisting schemas (Piaget and Inhelder, 1969/1969). Valdesolo et al. (2017, p. 3) claimed that “it is the accommodative component of the awe experience that distinguishes it from other epistemic emotions.” However, the process of restructuring cognition when new information contradicts prior knowledge or personal beliefs seems to be a common feature of all epistemic emotions, such as curiosity, interest, confusion, and awe (Campos et al., 2013; Vogl et al., 2019; see also Muis et al., 2018). Indeed, epistemic emotions are defined as emotions resulting from information-oriented appraisals about one's knowledge gap (see Silvia, 2010; Muis et al., 2018). Consequently, what distinguishes the awe experience from other related epistemic emotional experiences may not to be the need for accommodation but the physical or conceptual vastness of its elicitors (Keltner and Haidt, 2003; Campos et al., 2013). Moreover, recent findings demonstrated that awe is elicited by appraisals linked to exceeded expectancies rather than those that are only linked to disconfirmed expectancies; that is vastness would be central to awe while inconsistency would be peripheral to it (Gocłowska et al., 2021).

According to Keltner and Haidt (2003, p. 303), “vastness refers to anything that is experienced as being much larger than the self, or the self's ordinary level of experience or frame of reference.” Vastness can refer to physical, social, temporal, or any other conceptual dimension of an entity. Grand vista, natural phenomena, self-transcendent music, or monumental architecture are thus common stimuli of awe (Shiota et al., 2007; Ji et al., 2019; Negami and Ellard, 2021). Consequently, awe has been classified amongst self-transcendent emotions (i.e., emotions triggered by what is greater than the self and beyond its perceived boundaries; Shiota et al., 2014).

In order to catch the complexity of an emotion, it is relevant to focus both on its antecedents and consequences. During the last two decades, an increasing number of researchers have devoted their attention to highlight the effects of awe experiences on individuals. For example, awe has been found to diminish the sense of self (Bai et al., 2017; Sturm et al., 2020), and to promote spirituality (Saroglou et al., 2008; Van Cappellen and Saroglou, 2012), prosociality (Piff et al., 2015; Prade and Saroglou, 2016), humility (Stellar et al., 2018), environmentalism (Zhao et al., 2018), and well-being (Rudd et al., 2012; Bai et al., 2021). However, researchers who investigated awe, and how this emotion influence intraindividual and interpersonal changes, have primarily focused on the adult. Theoretical and empirical research on awe experiences during childhood is still dire missing. Yet, investigating awe in children may provide crucial information necessary for a better understanding of this emotion, its appraisals, consequences, and functions. Moreover, exploring how awe emerges and develops throughout childhood may have important implications for education, well-being, or recreational fields.

## Emergence and Development of Awe in Childhood

While cognitive accommodation is an innate learning process (Piaget and Inhelder, 1969/1969), the ability to perceive vastness presumably relies on the development of advanced cognitive skills therefore may not be present from birth. Vastness refers to what is perceived as being larger than the self. Thus, to experience vastness, children must presumably have the ability to form stable representations of the self and the world, then must be able to compare the stimulus to those representations. To perceive an entity as being greater than oneself presupposes having a sense of self.

Infants as young as 6 months old are able to detect a difference in the size of two stimuli (e.g., Brannon et al., 2006). However, detecting that one stimulus is bigger than another does not imply than it is perceived as vast. As mentioned, perceiving vastness presupposes a sense of self and self-evaluative processes skills. Based on the mirror self-recognition paradigm, most developmental and comparative psychologists consider that is not prior to ~18–24 months that children begin to show a sense of self, or self-awareness (for reviews on early self-awareness; see Diehl et al., 2011; Morin, 2021). At this age, however, toddlers only show physical self-awareness which remains relatively inconsistent during months; infants oscillate between self-recognition and the perception of seeing someone else facing them until ~4 years (see Rochat, 2003). As self-awareness is a prerequisite to the perception of vastness, first awe experiences could not appear before 18–24 months and would more probably emerge after 4 years; they may be slinking and exclusively triggered by physical vastness. During the early childhood, stimuli that provoke a need for cognitive accommodation should trigger epistemic emotions such as curiosity, interest, or uncanny, rather than awe since children would lack perception of vastness. Drawing very young children's attention on stimuli known to elicit awe, such as an awe-inspiring landscape, may yet be profitable for them because of the curiosity and interest that usually come along with this kind of stimuli. These more basic epistemic emotions have been shown to be strongly involved in knowledge exploration, explanation-seeking, and learning (see Muis et al., 2018; Vogl et al., 2019, 2020; Liquin and Lombrozo, 2020).

Awe experiences should progressively become more noticeable during middle childhood. From 4 to 7 years, self-awareness takes on more psychological manifestations. Abstract thinking and self-reflection abilities increase and may allow perception of conceptual (rather than only physical) vastness. Moreover, children of these ages progressively realize that significant others can evaluate them (see Harter, 2015). However, they cannot yet critically evaluate themselves and lack the cognitive ability to engage in social comparisons for self-evaluation purpose; holding an evaluation of the self and that of another in mind simultaneously in order to be compared exceed he cognitive ability of young children. Consequently, self-perception is unrealistically positive; children overestimate various of their competencies by focusing on virtuosity such as power and success (see Harter, 2006, 2015; Diehl et al., 2011). This normal manifestation of narcissism and egocentrism may limit the perception of vastness and so the emergence of awe experiences as well as their consequences (e.g., on humility and knowledge exploration).

Between the ages of 8 and 10, children show a more mature sense of self-continuity and of agency, and are able to form abstract and stable concepts. Narcissism and egocentrism gradually fade, and children become at times ashamed of the self. Moreover, cultural self, which refers to a sense of self as belonging to a larger community, expands during these years (Nelson, 2003). Finally, Children who are entering early adolescence show intense self-consciousness, greater introspection as well as self-reflection, and an increasing of social awareness. As cognitive and self-development progress, children should be more prone to experience awe. Indeed, in late childhood and early adolescence, children may perceive conceptual vastness and may be able to define themselves as belonging to larger entities, as well as to compare self-representations to the power or vastness of an abstract stimulus. At these ages, awe experiences may be particularly profitable for explanation-seeking and explorative behaviors underlying learning.

## Discussion

The present article aimed to qualify the assumptions of Valdesolo et al. (2017), who claimed that the accommodative component of the awe experience distinguishes it from other epistemic emotions, and that awe may be deeply involved in the process of early learning. In the first part, features of awe and its differences with epistemic emotions were examined. Two appraisals are needed to be met to experience awe: perceived vastness and a need for knowledge restructuring (Keltner and Haidt, 2003; Shiota et al., 2007; Campos et al., 2013). The need for knowledge restructuring seems to be a common feature of all epistemic emotions (Campos et al., 2013; Vogl et al., 2019; see also Muis et al., 2018), therefore what distinguishes the awe experience from other related epistemic emotional experiences is more probably the physical or conceptual vastness of its elicitors (Keltner and Haidt, 2003; Campos et al., 2013). Indeed, unlike curiosity or confusion, awe has been classified as a self-transcendent emotion, that is an emotion triggered by what is greater than the self and beyond its perceived boundaries (Shiota et al., 2014).

In the second part, we made conjectures about the emergence and development of awe in childhood. Perceiving something greater than the self presupposes stable representations of the self and the ability to compare the stimulus to those representations. In other words, awe should emerge and develop alongside with self-development. Consequently, in early childhood, stimuli that provoke a need for cognitive accommodation should trigger epistemic emotions such as curiosity, interest, or uncanny, rather than awe since young children lack self-awareness and self-reflection. Awe may, like other self-awareness dependent emotions, not emerge until 18–24 months. Indeed, early forms of embarrassment do not occur until 18–24 months, and more complex self-conscious emotions such as pride and shame would only emerge around 4 years, alongside with self-awareness and self-reflection development (for review see Tracy and Robins, 2004). Awe would thus be involved in the process of learning later in development than more basic epistemic emotions such as curiosity and interest.

Despite the arguments discussed in the present paper, to date no empirical evidence confirmed that the emergence and development of awe would depend on self-development. I hope researchers will grasp the important theoretical and practical implications of this topic and that empirical research on awe in childhood will be conducted. Recent experiments suggest that awe would exhibit distinct facial expression, non-verbal emotional vocalizations, and autonomic nervous system responding (Shiota et al., 2011; Campos et al., 2013; Cordaro et al., 2016) that could be used as cues to assess awe experiences in young children. It would also be interesting to investigate whether the consequences of awe are the same in adults and children. For example, awe has been found to promote prosociality and humility in adults (e.g., Prade and Saroglou, 2016; Stellar et al., 2018); this effect may be weaker in young children with high levels of narcissism and egocentrism (see Harter, 2015).

## Author Contributions

The author confirms being the sole contributor of this work and has approved it for publication.

## Conflict of Interest

The author declares that the research was conducted in the absence of any commercial or financial relationships that could be construed as a potential conflict of interest.

## Publisher's Note

All claims expressed in this article are solely those of the authors and do not necessarily represent those of their affiliated organizations, or those of the publisher, the editors and the reviewers. Any product that may be evaluated in this article, or claim that may be made by its manufacturer, is not guaranteed or endorsed by the publisher.
